# Enhancing vigilance for cerebral air embolism after pneumonectomy: a case report

**DOI:** 10.1186/s12890-020-01358-6

**Published:** 2021-01-07

**Authors:** Yijun Mo, Lina Lin, Jun Yan, Chenghua Zhong, Jun Kuang, Quanwei Guo, Dongfang Li, Mengxi Wu, Zesen Sui, Jianhua Zhang

**Affiliations:** 1 Department of Thoracic Surgery, Shenzhen Hospital, Southern Medical University, No.1333 Xinhu Road, Baoan District, Shenzhen, 518101 Guangdong China; 2 School of Nursing, Xinhua College of Sun Yat-Sen University, No. 19 Huamei Road, Guangzhou, 510520 Guangdong China

**Keywords:** Cerebral air embolism, Pneumonectomy, Neurological recovery

## Abstract

**Background:**

Vascular air embolism (VAE) is a rare but important complication that has not been paid enough attention to in the medical process such as surgery and anesthesia.

**Case presentation:**

We report for the first time that a 54-year-old male patient with central lung cancer developed severe complications of CAE after right pneumonectomy. After targeted first-aid measures such as assisted breathing, mannitol dehydration and antibiotic treatment, the patient gradually improved. The patient became conscious at discharge after 25 days of treatment but left limb was left with nerve injury symptoms.

**Conclusion:**

We analyzed the possible causes of CAE in this case, and the findings from this report would be highly useful as a reference to clinicians.

## Background

Vascular air embolism (VAE) is a rare but important complication that has not been paid enough attention to in the medical process such as surgery and anesthesia [1]. Cerebral air embolism (CAE) can lead to insufficient blood perfusion in the central nervous system, cerebral ischemia and hypoxia, which can cause severe brain edema, with a high fatality rate [2]. In the field of thoracic surgery, pulmonary fine needle aspiration biopsy, thoracic penetrating injury, etc. can lead to the occurrence of CAE [3, 4]. In this article, we report for the first time a serious complication of CAE after pneumonectomy.

## Case presentation

**Fig. 1:**
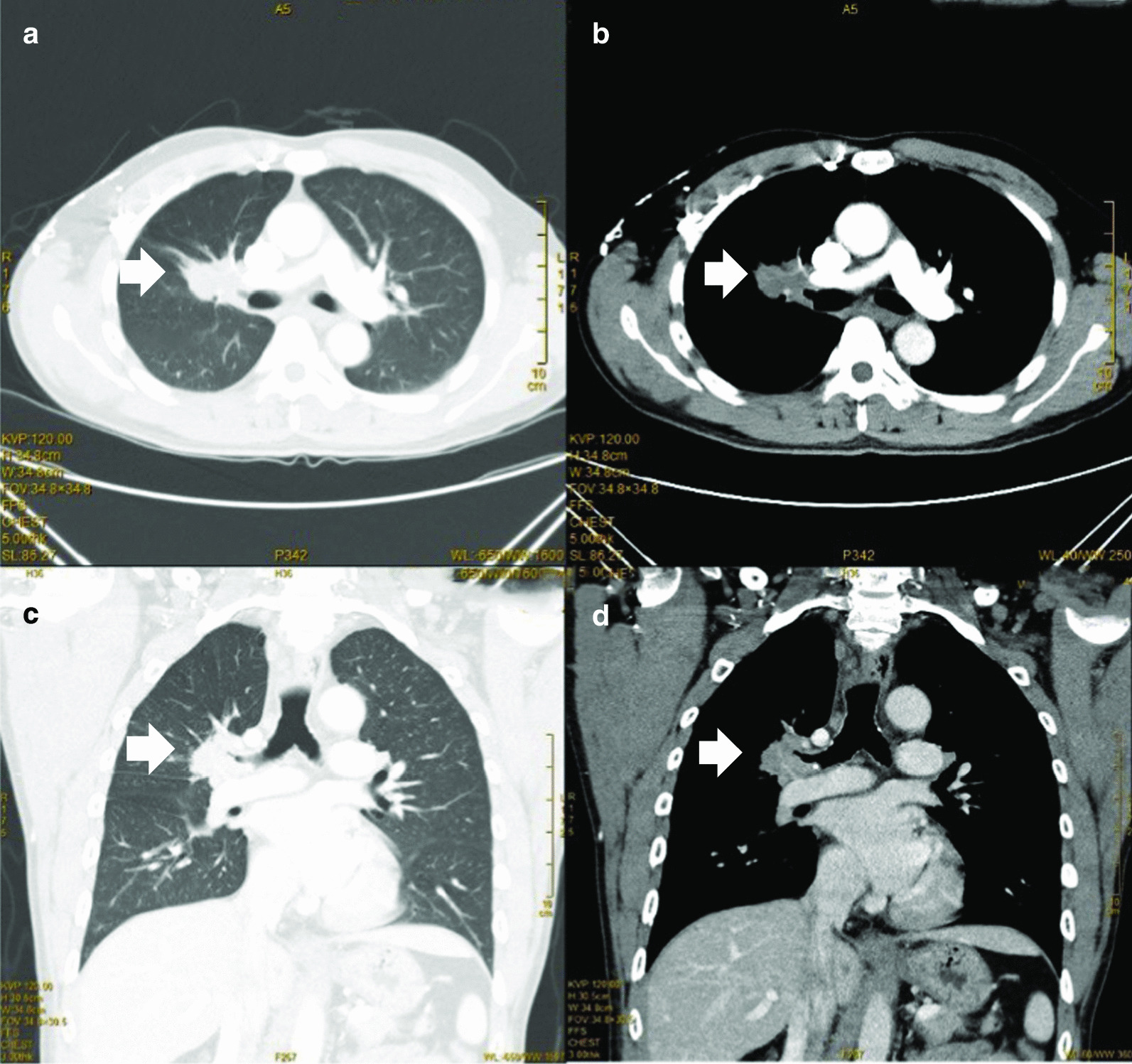
Chest CT of right central lung cancer. **a**. Cross-sectional, lung window showed right central lung cancer. **b**. Mediastinal window, tumor invaded right main bronchus and right pulmonary artery trunk. **c d**. Frontal plane, right central lung cancer invade right main bronchus and right pulmonary artery trunk

A 54-year-old man diagnosed with locally advanced central squamous cell carcinoma was hospitalized in our hospital. The patient had cough and shortness of breath for 3 months. Chest CT showed central lung cancer in the upper lobe of the right lung, with tumor invading the trunk of the right pulmonary artery, the main bronchus and the upper lobe bronchus (Fig. 1). Squamous cell carcinoma was confirmed by further bronchoscopy. Three courses of neoadjuvant chemotherapy were performed before surgery. The patient refused to continue chemotherapy and asked for active surgical treatment. According to the TNM staging system, the clinical stage of this tumor is cT4N1M0.

**Fig. 2:**
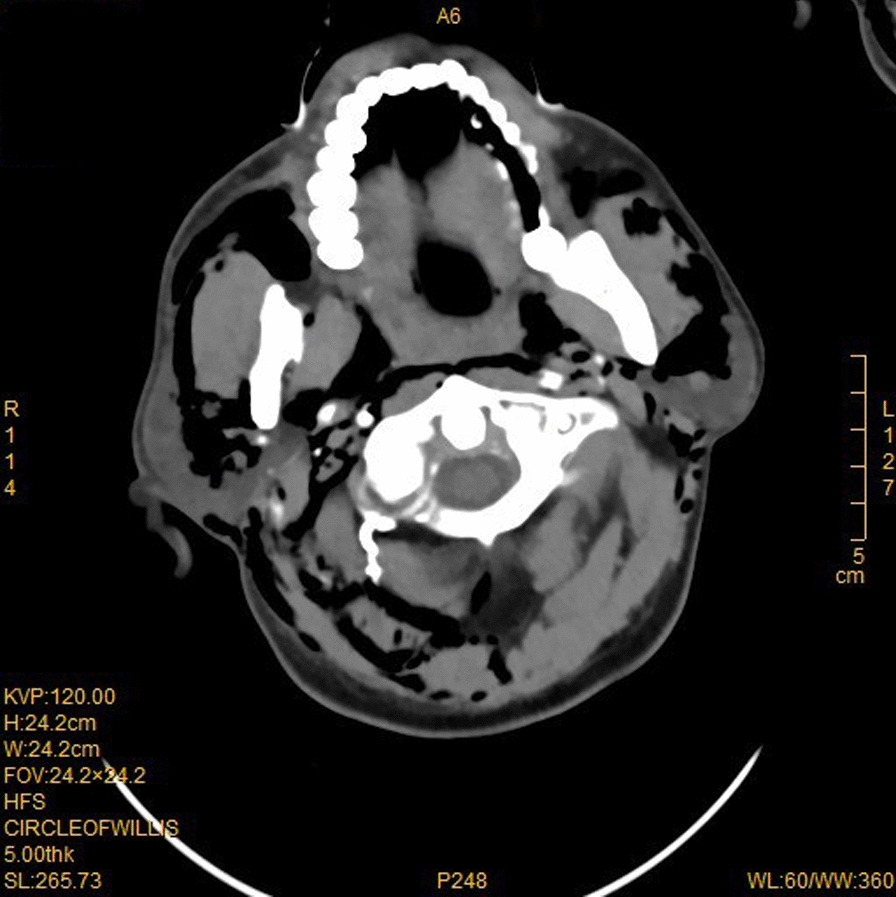
A large amount of gas is present in the interstitial space of the neck

**Fig. 3:**
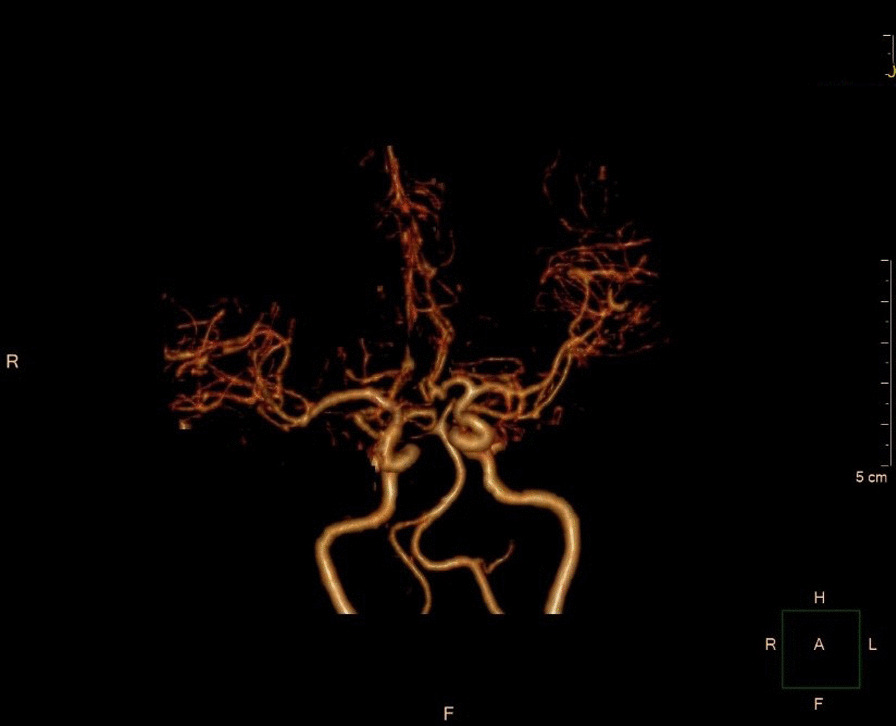
CTA results showed that there were no abnormalities such as embolism in the main cerebral artery

**Fig. 4:**
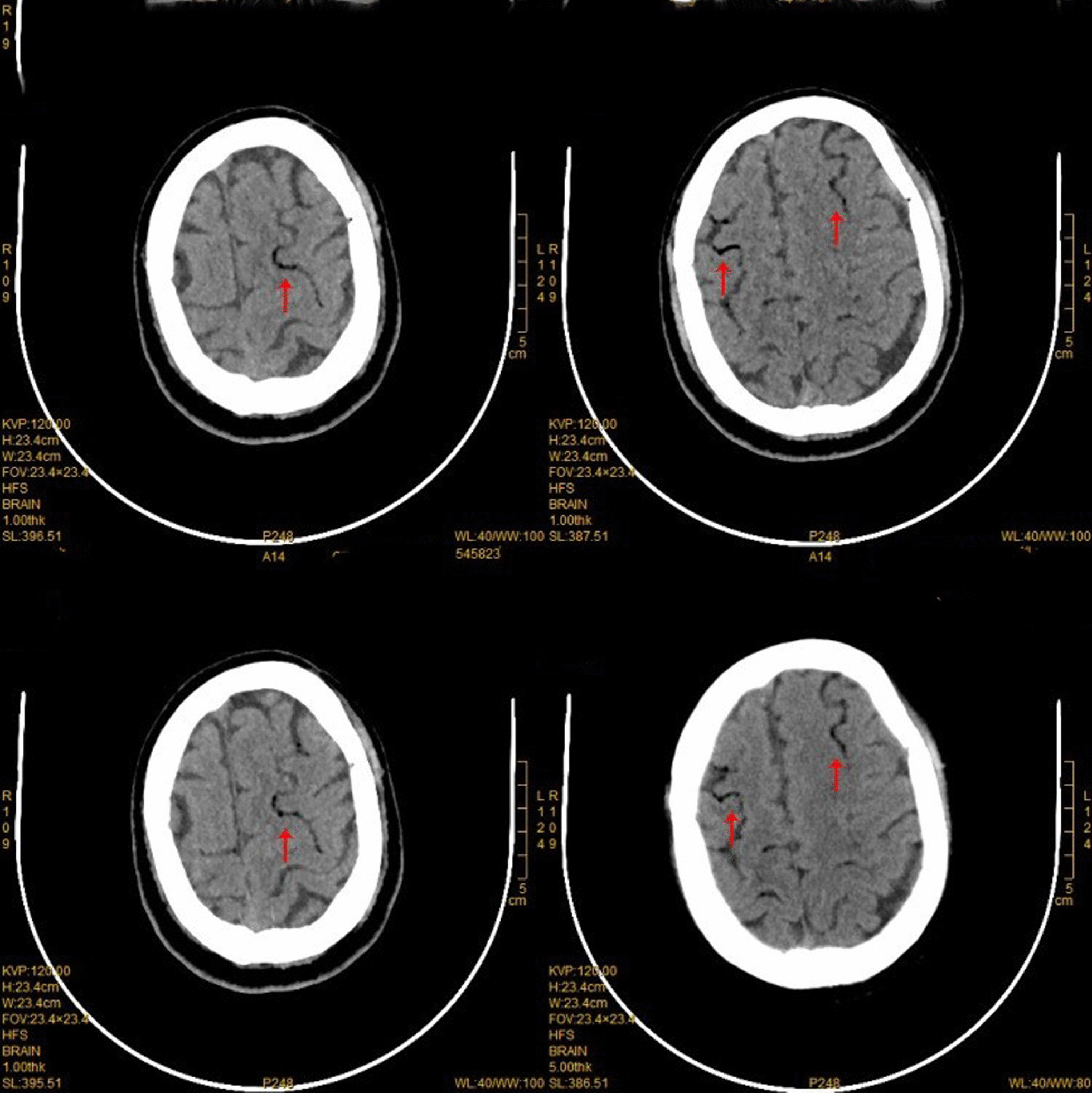
Multiple free air can be seen in the blood vessels of bilateral frontal sulcus as indicated by the arrows

**Fig. 5:**
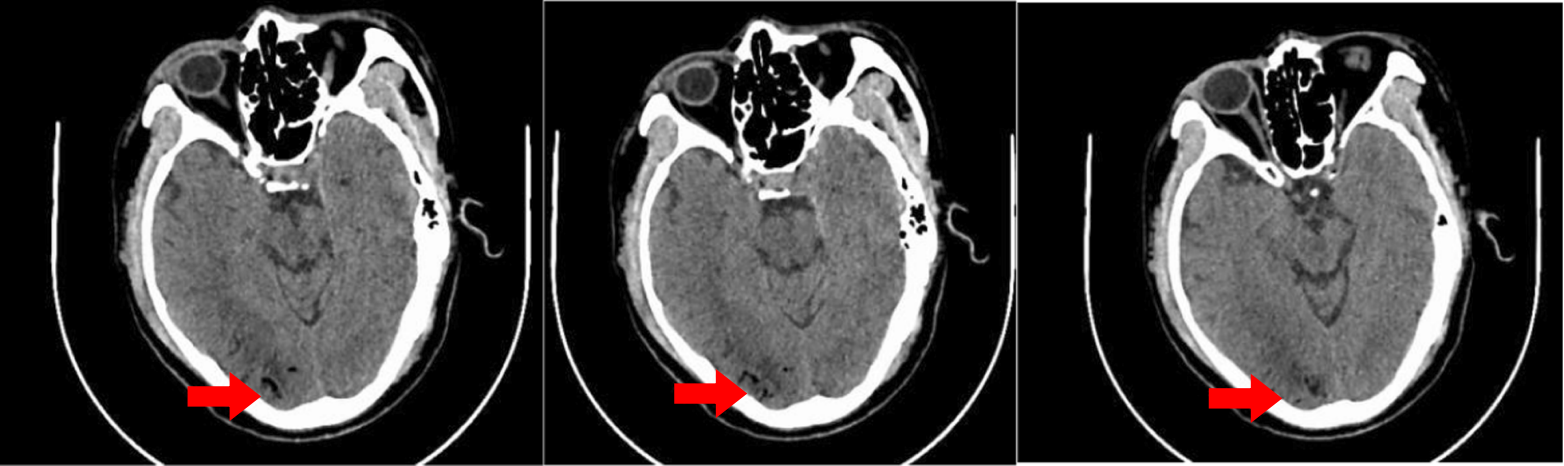
Dispersed free air was seen in the vessels of the right occipital lobe and suspicious cerebral infarction lesions were seen

**Fig. 6:**
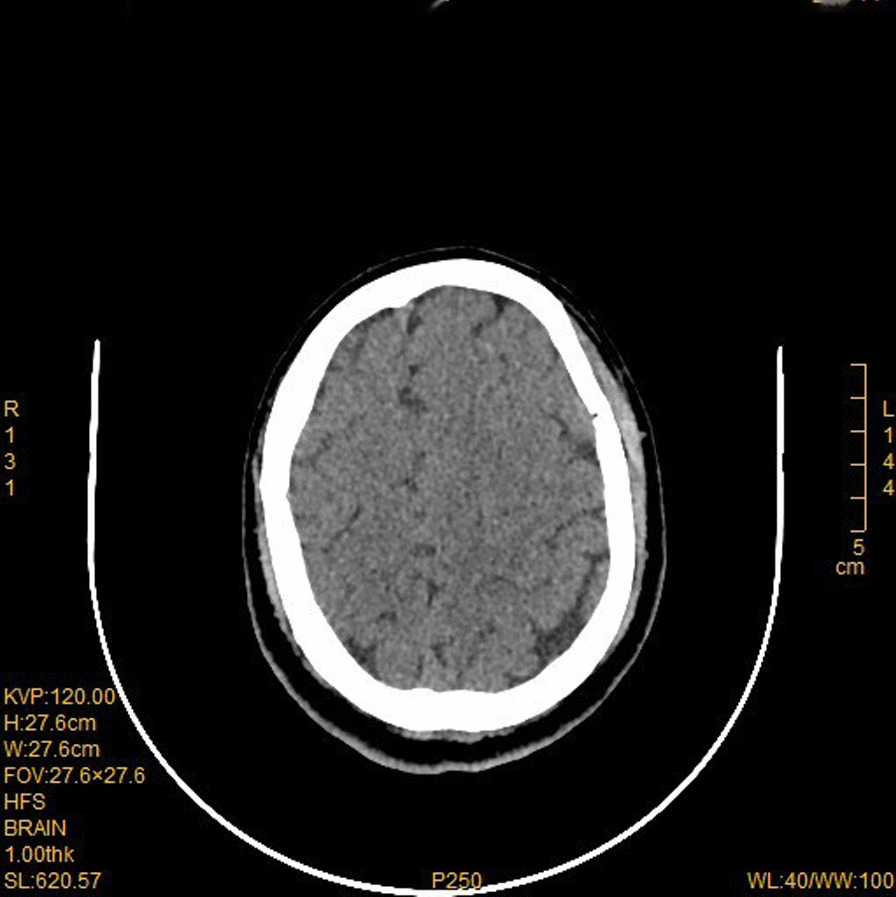
Significant reduction in cerebral air embolism 24 h after treatment

**Fig. 7:**
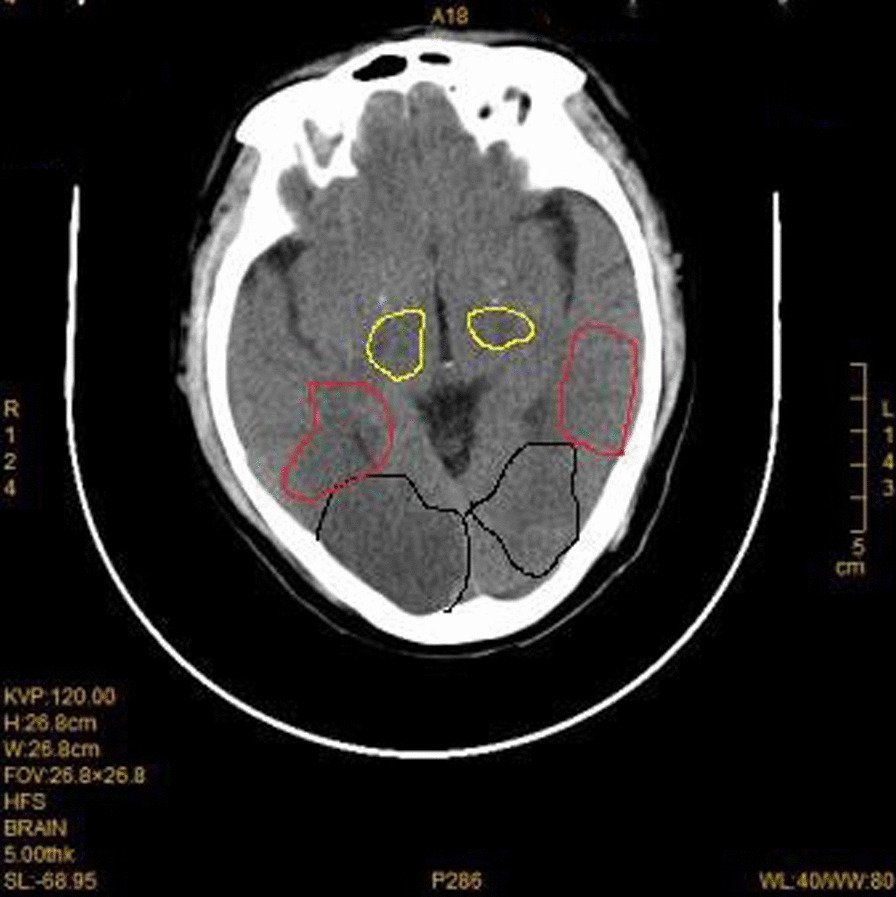
Three days after air embolism, CT showed a patchy low-density shadow in bilateral thalamic basal ganglia (yellow area), temporal lobe (red area) and occipital lobe (black area), which was the cerebral infarction lesion after air embolism

General anesthesia was induced and maintained according to standard protocols during surgical preparation. Then right pneumonectomy was performed, and the surgical incision was located at the posterolateral side of the fourth intercostal space. The pulmonary artery, pulmonary vein and bronchus were cut off with a stapler, with intraoperative bleeding of about 100 ml. After the operation, the thoracic drainage tube was clamped, and the patient returned to the ward after waking up. Three hours after the operation was completed, the patient suddenly lost consciousness while sitting in bed chatting with his wife. The patient’s blood pressure was low (76/53 mmHg), and the indexes of heart rate, respiration and oxygen saturation were normal. After opening the thoracic drainage tube, a small amount of tension gas and 200 ml of bloody fluid was discharged. We immediately performed CT examination and excluded brain lesions. CT examination showed that there was a large amount of gas in the tissue space between the chest and neck (Fig. 2). Blood clots accumulated in the thoracic cavity, and CT angiography showed no abnormalities in the major cerebral arteries (Fig. 3). However, multiple free air can be seen in the blood vessels of bilateral frontal sulcus (Fig. 4). In addition to free air, suspicious cerebral infarction was also seen in the right occipital lobe (Fig. 5). It is presumed that air entering the cerebral circulation led to air embolism. The patient had seizures soon after the CT scan, manifesting as binocular gaze and tremor of limbs. Then the patient was quickly transferred to ICU. Respiratory assistance, mannitol dehydration and empiric antibiotic treatment were used after ICU transfer. 800 ml pleural fluid was drained from the thoracic cavity 6 h after the operation, and a second thoracotomy was performed to stop bleeding. Then blood clot was removed from the thoracic cavity. After the operation, the patient was sent back to the ICU for ventilator-assisted breathing, and the head was protected by mild hypothermia using an ice blanket to prevent excessive brain damage. At the same time, mannitol dehydration was used to reduce brain edema and anti-infection treatment was carried out. On the next day, the brain CT showed a significant decrease of air in the brain (Fig. 6). There was no significant increase in cerebral infarction lesions compared with the first day. After 3 days of ventilator-assisted breathing, the patient’s condition gradually improved. On the third day after air embolism, the brain CT was reexamined. There were patchy low density areas in bilateral thalamic basal ganglia, temporal lobe and occipital lobe, which was cerebral infarction lesions. The patient was discharged after 25 days of treatment after cerebral infarction. He was conscious at the time of discharge, with neurological impairment symptoms of bilateral in which symptoms of left limb are more serious. The process from onset to recovery was smooth. After 3 months of follow-up, most of the neurological deficit symptoms had been recovered except for the left upper limb (Fig. 7).

## Discussion

In the present case, the patient had sudden change of consciousness after surgery. CT images showed a large amount of gas in the interstitial space between the neck and thorax. We speculated that the pleural drainage fluid increased continuously after surgery while the thoracic drainage tube was clamped and the gas could not be discharged out of the body, resulting in the occurrence of right tension pneumothorax. The presence of tension pneumothorax was evidenced by tension gas drainage when the patient was unconscious. Because there are a large number of open vascular beds in the thorax of lung cancer surgery, air enters the venous system when the intrathoracic pressure exceeds the venous pressure. Gas emboli entering the vein may shunt into the arterial circulation through an intrapulmonary arteriovenous fistula. Another possibility is that the gas entering the blood vessel exceeded the lung’s filtration capacity, causing the gas from the venous side to enter the arterial side and form paradoxical air embolism, which eventually leads to cerebral air embolism [5–8]. Some rare case reports regarding lung surgery of wedge resection and segmentectomy has been associated with cerebral air embolism [9, 10]. It is also possible that air enters the arterial circulation through a backward left shunt of the heart. However, preoperative color Doppler echocardiography showed no cardiac changes, so we excluded the cardiac shunt pathway. When air bubbles reach the brain tissue, they activate neutrophils, promote blood stasis, and eventually lead to cerebral infarction. The clinical presentation of cerebral air embolism is determined by the quantity of gas and the areas of the brain that are affected, ranging from minor motor weakness and headache to convulsion, loss of consciousness and coma [2, 11].

Oxygen should be provided to patients to the maximum extent after CAE, which can reduce the volume of gas emboli. Hyperbaric oxygen therapy was reported to be an effective method in promoting the prognosis of CAE [8, 11–13]. Our patient had no respiratory failure symptoms after CAE, but we still used ventilator to assist breathing for 3 days, which should be an effective way to provide sufficient oxygen to the patient’s brain. Reexamination of CT results the next day demonstrated a significant reduction of air in the cerebral circulation. Patients’ outcome after air emboli can be variable and may depend on initial presentation. In one series of patients presenting with air emboli, 50% of patients presenting without encephalopathy had good or complete recovery while only 29% of patients presenting with encephalopathy had good recovery [14].

The best treatment strategy for CAE is early recognition and prevention [15]. For example, if patients have signs of impaired nervous system function such as altered consciousness and hemiplegia after pulmonary surgery, the possibility of air embolism should be taken into consideration. Early craniocerebral CT, MRI and other imaging examinations should be performed [16], and appropriate treatment options should be selected according to the condition, which is essential in saving lives and reducing the disability rate. It is recommended that thoracic drainage after pneumonectomy should be connected to a water-seal balanced system to avoid mediastinal shift and allow the appropriate drainage of fluid and air. In summary, this current reported case suggested that patients with neurological disorders after pulmonary surgery need to be vigilant about the occurrence of CAE.

## Data Availability

The datasets used and/or analyzed during the current study are available from the corresponding author on reasonable request.

## Abbreviations

CAE: Cerebral air embolism
ICU: Intensive Care Unit
VAE: Vascular air embolism
